# Differential mitochondrial priming by BCR::ABL1 in B-cell precursor acute lymphoblastic leukemia

**DOI:** 10.1038/s41375-026-02944-z

**Published:** 2026-03-30

**Authors:** Hanna Kirchhoff, Caroline Schoenherr, Lisa Fleischer, Steven R. Talbot, Florian H. Heidel, Anke K. Bergmann, Matthias Eder, Michaela Scherr

**Affiliations:** 1 Department of Hematology, Hemostasis, Oncology and Stem Cell Transplantation, Hannover Medical School, Hannover, Germany; 2 Institute for Laboratory Animal Science, Hannover Medical School, Hannover, Germany; 3 Clinical Genetics and Genomic Medicine, University Hospital Würzburg, Würzburg, Germany; 4 Department of Human Genetics, Hannover Medical School, Hannover, Germany

**Keywords:** Leukaemia, Leukaemia

## To the Editor

Despite targeted therapies including tyrosine kinase inhibitors (TKI) and immune effector cell-based approaches, BCR::ABL1^+^ acute lymphoblastic leukemia (ALL) remains a high-risk disease with allogeneic stem cell transplantation considered the best-established curative option. However, due to the age-dependency of BCR::ABL1^+^ ALL incidence and enhanced vulnerability of elderly patients, optimization of drug-based therapies remains an urgent clinical need [1, 2].

BH3 mimetics induce apoptosis by releasing activator BH3-only proteins (e.g., BIM, BID) from multidomain anti-apoptotic BCL2 family members (e.g., BCL2, BCLXL, MCL1), thereby inducing oligomerization of effector molecules (BAK/BAX) and mitochondrial outer membrane permeabilization (MOMP) [3]. Differential complex formation of pro- and anti-apoptotic proteins may prime mitochondria, thereby modulating sensitivity towards apoptosis induction. Using primary and patient-derived xenograft (PDX) BCP-ALL cells, we here describe differential mitochondrial priming and BCLXL dependency in BCR::ABL1^+^ ALL, allowing rapid diagnostics and optimization of targeted therapies.

**Fig. 1:**
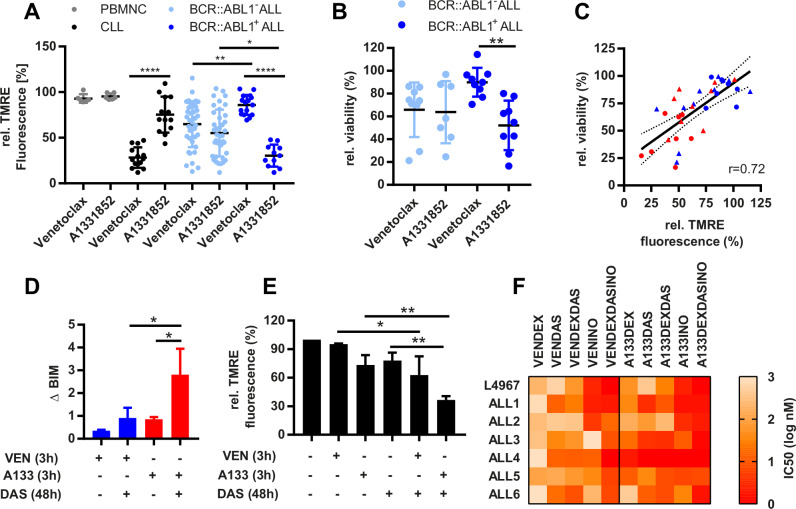
BCR::ABL1^+^ ALL cells are sensitive to BCLXL inhibition by A1331852. **A** Relative TMRE fluorescence, reflecting mitochondrial membrane potential in a short-term BH3 mimetics assay with primary samples. PBMNC *n* = 6, CLL *n* = 14, BCR::ABL1^-^ ALL *n* = 38; BCR::ABL1^+^ ALL *n* = 16. **B** Cell viability of primary or PDX BCR::ABL1^-^ (*n* = 9) or BCR::ABL1^+^ ALL (*n* = 9) in co-culture with human MSC after 48 h incubation with 100 nM Venetoclax or A1331852, respectively assessed by PI/Calcein AM staining. **C** Correlation of cell viability with short-term changes in TMRE fluorescence. Pearson correlation *r* = 0.72. Red and blue symbols indicate treatment with A1331852 and Venetoclax, respectively, of BCR::ABL1^+^ ALL (circles) and BCR::ABL1^-^ ALL samples (triangles). **D** Densitometric analysis of BCL2 and BCLXL immunoprecipitations (mean of three BCR::ABL1^+^ ALL PDX models) and **E** mean TMRE fluorescence intensity of three BCR::ABL1^+^ ALL PDX models treated for 48 h with 50 nM Dasatinib, followed by 3-hour treatment with Venetoclax (1 µM) or A1331852 (100 nM). **F** IC50 of 7 primary or PDX (L4967) samples in MSC co-culture treated for 48 h with the indicated drug combinations in increasing concentrations in fixed ratios. IC50 refers to the respective BH3 mimetic in the drug combinations. *p < 0.05, **p < 0.01, ****p < 0.0001 indicate statistical significance calculated using one-way ANOVA with Bonferroni post hoc test (**A**, **B**, **D** and **E**).

Primary BCP-ALL and CLL samples (*n* = 68) (Supplementary Table 1, 2) were profiled for BCL2- (Venetoclax) and BCLXL-specific (A1331852) MOMP induction by direct staining of viable cells with tetramethylrhodamine ethyl ester (TMRE) as previously reported [4, 5]. This approach avoids any impact of cell permeabilization on mitochondrial membrane potential ΔΨ_m_ and cell viability [6]. In CLL, Venetoclax induced strong TMRE destaining, whereas normal peripheral blood mononuclear cells (PBMNC) were unaffected by both BH3 mimetics. In BCP-ALL, TMRE destaining induced by Venetoclax and A1331852 was heterogeneous with no differential sensitivity in BCR::ABL1^-^ samples (Fig. 1A). In contrast, A1331852 induced potent TMRE destaining in all BCR::ABL1^+^ ALL samples tested, while Venetoclax had almost no effect. This pattern was reflected in 48-hour BCP-ALL cultures (*n* = 18) on primary mesenchymal stroma cells (MSC) with strong A1331852 cytotoxicity but minimal effects by Venetoclax (Fig. 1B). No such difference was observed in BCR::ABL1^-^ samples. Importantly, TMRE destaining correlated to 48-hour cell viability for both BH3 mimetics in both BCR::ABL1^+^ and BCR::ABL1^-^ BCP-ALL, with reduced TMRE fluorescence predicting reduced cell survival (Fig. 1C).

Since BIM has been identified as uniform binding partner of BCLXL and BCL2 in primary BCP-ALL cells [4, 7, 8] we analyzed BCR::ABL1-dependent BIM complex formation with BCL2 and BCLXL in primary PDX BCR::ABL1^+^ and BCR::ABL1^-^ cells by immunoprecipitation and immunoblotting. BIM expression was reduced in BCR::ABL1^+^ as compared to BCR::ABL1^-^ cells (Supplementary Fig. 1A, B) as reported earlier [4, 9]. BCL2 and BCLXL expression was heterogeneous in both groups; however, BIM immunoprecipitates revealed preferential BIM/BCLXL complex formation only in BCR::ABL1^+^ cells, while no such difference was found for BIM/BCL2 complexes (Supplementary Fig. 1A, C, D). These data indicate that despite reduced expression, BIM preferentially complexed with BCLXL in BCR::ABL1^+^ BCP-ALL.

We next analyzed BIM release from BCL2/BIM and BCLXL/BIM complexes by Venetoclax and A1331852 in three BCR::ABL1^+^ BCP-ALL PDX models (Supplementary Fig. 2A–E). Venetoclax interrupted BCL2/BIM complexes to 55.6% after 5 h (Supplementary Fig. 2B, C), whereas A1331852 induced faster and stronger complex dissociation, leaving only 16.5% intact (Supplementary Fig. 2D, E). In contrast, in a BCR::ABL1^-^ ALL model, Venetoclax was more effective than A1331852 (Supplementary Fig. 2F, G). These complex dissociation patterns corresponded to TMRE destaining kinetics (Supplementary Fig. 2H–K), demonstrating enhanced BCLXL/BIM complex dissociation, MOMP induction and augmented cytotoxicity by A1331852 specifically in BCR::ABL1^+^ ALL.

In addition, we analyzed mitochondrial priming depending on BCR::ABL1 tyrosine kinase activity in three BCR::ABL1^+^ ALL PDX models (Supplementary Fig. 3). Consistently, 1 µM Venetoclax induced only minor BCL2/BIM dissociation (left, upper panels), whereas 100 nM A1331852 effectively disrupted BCLXL/BIM complexes (left, lower panels) (Supplementary Fig. 3A). Following 48-hour preincubation with Dasatinib, this differential complex dissociation remained unaffected. However, densitometric quantification of total BIM released by A1331852, considering enhanced BIM expression upon TKI treatment, revealed a nearly 3.3-fold increase (Fig. 1D and Supplementary Fig. 3C, D). Venetoclax also promoted increased, albeit substantially weaker, BIM release from BCL2/BIM complexes upon Dasatinib treatment. As expected, BIM release corresponds to MOMP induction (Fig. 1E). Finally, synergy with Dasatinib, Dexamethasone and Inotuzumab was stronger for A1331852 than for Venetoclax as assessed by IC50-based cytotoxicity and combination indices in BCR::ABL1^+^ primary or PDX samples (Fig. 1F and Supplementary Fig. 4A), while BCR::ABL1^-^ PDX samples showed no preferential A1331852 sensitivity (Supplementary Fig. 4B, C)

**Fig. 2:**
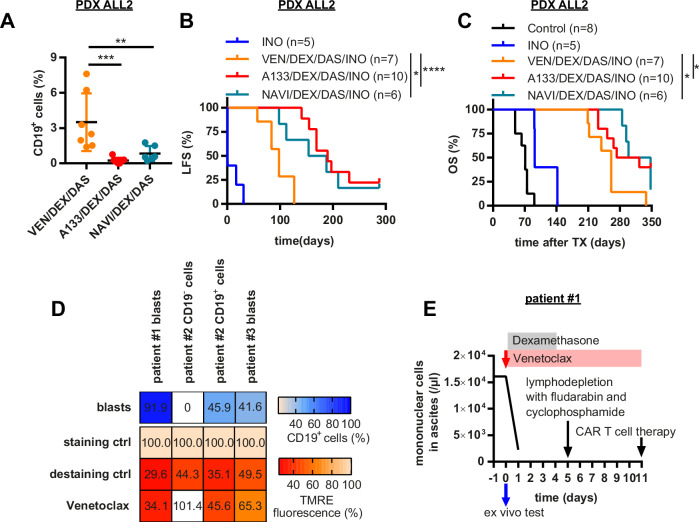
DEX/DAS/INO combinations with BCLXL-targeting BH3 mimetics are superior to Venetoclax combination therapies in BCR::ABL1^+^ PDX models in vivo. NSG mice were transplanted intravenously with PDX ALL2 cells **A** Human CD19^+^ cells in the peripheral blood of PDX ALL2 mice after two weeks of treatment with VEN/DEX/DAS and A133/DEX/DAS, respectively. **B** Leukemia-free survival (LFS) and **C** overall survival (OS) of ALL2 mice treated with INO alone, or with the combinations VEN/DEX/DAS/INO or A133/DEX/DAS/INO, respectively. **D** CD19^+^ blast cells in patient samples and relative TMRE fluorescence, reflecting mitochondrial membrane potential in a short-term assay for Venetoclax sensitivity. Untreated cells served as staining control. Treatment with the ionophore FCCP (5 µM) for 20 min was used as destaining control **E** Therapy schedule, and clinical response of patient #1. *p < 0.05, **p < 0.01, ***p < 0.001, ****p < 0.0001 indicate statistical significance calculated using **A** one-way ANOVA with Bonferroni post-hoc test or **B**, **C** log-rank test.

Subsequently we analyzed differential BH3 mimetic-based therapies in combination with synergizing compounds in vivo in two BCR::ABL1^+^ ALL and one lymphatic blast crisis (BC) PDX mouse model. Experiments comparing simultaneous and successive applications of Inotuzumab in combination with Dasatinib, Dexamethasone with or without Venetoclax revealed a significant survival benefit by Venetoclax but no difference by the Inotuzumab application sequence (Supplementary Fig. 5A). For all subsequent in vivo experiments, we therefore used Dasatinib, Dexamethasone and Venetoclax vs. A1331852 (or Navitoclax), respectively, as induction followed by Inotuzumab application. Treatment was started after confirmed engraftment (Supplementary Fig. 5B–D). In both PDX ALL1 and PDX ALL2 models, A1331852 outperformed Venetoclax in remission induction after two weeks of treatment based on bioluminescence in vivo imaging (BLI) (Supplementary Fig. 5E, F) and huCD19^+^ ALL cell detection in peripheral blood (Fig. 2A), respectively. This superiority of A1331852 also translated into longer leukemia-free (mean 64 and 48 days in PDX ALL1, 180 and 98 days in PDX ALL2) and overall survival (mean 198 and 162 days in PDX ALL1, 323 and 261 days in PDX ALL2) (Fig. 2B, C and Supplementary Fig. 5G, H) with 4/9 (44.4%) PDX ALL2 mice surviving the one-year observation period (2/9 mice without detection of ALL upon post-mortem analysis). The CML BC model reached the termination criteria with the death of the last mouse in the Venetoclax group (Supplementary Fig. 6A–E). At that time, 4/6 mice in the A1331852 group were alive, and final post-mortem analysis revealed significantly lower spleen weight and reduced huCD19^+^ ALL cells in bone marrow and spleen (Supplementary Fig. 6C–E). Additionally, we analyzed Navitoclax targeting both BCL2 and BCLXL, in comparison to A1331852, in the PDX ALL2 model. Navitoclax was equally effective as A1331852 for MOMP induction in primary BCR::ABL1^+^ ALL cells (Supplementary Fig. 7) and equally outperformed Venetoclax in terms of leukemia-free (190 and 171 days compared to 98 days) and overall survival (298 and 323.5 compared to 261 days, with 1/6 mice without leukemia detection upon termination) (Fig. 2B, C).

Finally, we used ex vivo MOMP induction and mitochondrial priming for Venetoclax-based personalized therapies for individual r/r ALL and lymphatic BC CML patients beyond established therapy. The first r/r BCP-ALL patient (#1) presented with painful ascites with a homogenous blast cell population responsive to Venetoclax (Fig. 2D and Supplementary Fig. 8A). Upon treatment with Venetoclax and Dexamethasone, ascetic blasts cleared very rapidly (Fig. 2E), and lymphodepletion for subsequent CAR T cell therapy was started within 5 days. The second patient (#2) presented with r/r BCP-ALL after allogeneic transplantation and CAR T cell therapy. Bone marrow showed about 45% blast cell infiltration and CD19^-^ and a CD19^+^ cell populations, with only the latter showing sensitivity to Venetoclax (Fig. 2D and middle, Supplementary Fig. 8B). The patient was treated with Inotuzumab and Venetoclax. Bone marrow infiltration rapidly cleared after two Inotuzumab applications to flow cytometry-based MRD-negativity after the third dose of Inotuzumab, and the patient underwent secondary allogeneic stem cell transplantation (Supplementary Fig. 8C). The third patient (#3) relapsed after allogeneic stem cell transplantation for lymphoid CML BC with T315I mutation with poor response to Ponatinib (Supplementary Fig. 8D). Venetoclax induced MOMP in CD19^+^ blast cells (65.3%) (Fig. 2D, right), and treatment with Venetoclax, Ponatinib, Dexamethasone and Inotuzumab rapidly induced cytologic (Supplementary Fig. 8E) and major molecular remission (BCR::ABL1 dropping from 3.98 to 0.007 on day 27 after treatment start). Finally, ex vivo analysis of mitochondrial priming induced by the respective combination therapies confirmed increased MOMP induction upon Venetoclax treatment in all three patients (Supplementary Fig. 8F–H).

Our study points to two clinically relevant issues: First, BCR::ABL1^+^ ALL can be detected by functional flow cytometry-based testing for MOMP induction feasible in parallel with diagnostic immunophenotyping. BH3 mimetic responses in intact, non-permeabilized viable cells can identify differential sensitivities, with MOMP induction predicting cytotoxicity and clinical endpoints in preclinical PDX models. This may be used for personalized bridging therapy in selected r/r BCP-ALL patients. Second, we identify BCLXL as a promising target for therapeutic intervention in BCR::ABL1^+^ ALL. A1331852 releases BIM from BCLXL/BIM complexes, and the absolute amount of BIM release increases by inhibition of BCR::ABL1 tyrosine kinase activity. TKIs (and Dexamethasone) increase BIM expression, preferentially binding to BCLXL and inducing mitochondrial priming as well as drug synergy with BH3 mimetics. Currently, no BCLXL BH3 mimetic is clinically available due to thrombocytopenia [10–12] and significant cardiovascular A1331852 toxicity in preclinical large animal models [13]. However, we observed only mild and fully reversible side effects in A1331852-treated mice. The use of Navitoclax [14], pulsatile application schedules [4] or platelet-sparing targeted BCLXL degradation [15] may limit systemic toxicity of BCLXL-targeting and allow detailed analyses of BCLXL-directed therapy in combination with ABL-specific kinase inhibition in BCR::ABL1^+^ ALL. Finally, Inotuzumab and immune effector cell-based therapies, such as Blinatumomab, may provide additional components for a mechanism-based and ultimately curative drug therapy for BCR::ABL1^+^ ALL.

## Supplementary information

Supplement

## Data Availability

For original data, please contact eder.matthias@mh-hannover.de.
