# Supplementary material for: Differential mitochondrial priming by BCR::ABL1 in B-cell precursor acute lymphoblastic leukemia
Source: Leukemia. 2026 Mar 30;40(5):1076–9. doi: 10.1038/s41375-026-02944-z (PMC13148979; doi:10.1038/s41375-026-02944-z)
Supplement: Supplementary file 1 — Supplement [file 41375_2026_2944_MOESM1_ESM.docx]

**Supplement**

**Supplemental Methods:**

*Cell culture*

RS4;11 cells (DSMZ, Braunschweig, Germany) were cultured in a cell density of 1x10^6^/ml in RMPI 1640 supplemented with 10% FCS and 1% Penicillin/Streptomycin.

*Pharmacological agents*

Venetoclax, Navitoclax (both Selleck Chemicals), A1331852 (ApexBio), and Dasatinib (Santa Cruz Biotechnology) were solubilized in DMSO, and Dexamethasone (Selleck Chemicals) was solubilized in PBS at stock concentrations of 10 mM, respectively. Inotuzumab-ozogamicin (Pfizer) 0.25 mg/ml stocks were prepared in 0.9% NaCl.

For *in vivo* studies, Venetoclax, Navitoclax and A1331852 were solubilized in a vehicle consisting of 60% Phosal, 30% PEG400, and 10% Ethanol. Dexamethasone and Dasatinib were solubilized in 0.1 M sodium citrate, pH 5.2, at the respective concentrations.

*Immunoprecipitation and Immunoblotting*

PDX cells were freshly isolated from end-stage leukemic NSG mice and treated with BH3 mimetics (1 µM) for 1-5 hours or for 48 hours with Dasatinib (50 nM), followed by 3 hours of incubation with BH3 mimetics (100 nM A1331852 or 1 µM Venetoclax). Cells were pelleted by centrifugation (5 min, 1300 rpm) and pellets were resuspended in CHAPS buffer (50 mM Tris-HCl [pH 7.4], 150 mM NaCl, 1 mM EGTA, 1 mM EDTA, 1% CHAPS) supplemented with mini complete protease inhibitor cocktail and PhosStop phosphatase inhibitor cocktail (both Roche diagnostics, Mannheim, Germany) on ice for 30 min. Immunoprecipitation was performed as described earlier with anti-BCL2 antibody (05-826, Millipore), anti-BCLXL (2762, Cell Signaling Technology) or anti-BIM (2819, Cell Signaling Technology)) precoupled to magnetic Dynabeads protein G beads (Invitrogen) at 4°C. After 20 hours immunocomplexes were recovered, washed in PBS supplemented with protease and phosphatase inhibitors (Roche), and resuspended in sample buffer.

Subsequently immunoblot analysis was performed as described earlier (21) using the following antibodies for detection: anti-BIM (cs2933), anti-BAX (cs2772), anti-BAK (cs3814), anti-BCLXL (cs2762) (all Cell Signaling Technology), anti-Cox IV (33985, abcam) and anti-BCL2 (551051, Becton Dickinson). For BIM immunoprecipitations Ponceau staining was performed as loading control.

Quantitative densitometric analysis of co-immunoprecipitations was performed by chemiluminescence imaging of immunoblots using ChemiDoc MP Imaging system and Image Lab software version 5.0 (both Bio-Rad). The intensity ratio of the protein of interest band to the band of the respective precipitated protein was calculated to measure changes in protein levels. For BIM the isoform BIM_EL_ was quantitated.

*Patient material and TMRE Assay*

BM and PB samples were collected from newly diagnosed or relapsed CLL and B-lineage ALL patients provided from either Hannover Medical School or hospitals affiliated to the GMALL study group (Suppl. Tables 1 and 2). Written informed consent was obtained from all patients in accordance with the Declaration of Helsinki and the Ethics Committee of Hannover Medical School (8345_BO_S_2019). Primary cells were isolated by Ficoll-gradient density centrifugation and subsequent lysis of erythrocytes by ammonium chloride (0.83%). Cells were incubated at a cell density of 1x10^6^ cells /ml in RPMI, 10% FCS, 1% P/S, and incubated with 1 µM of the respective BH3 mimetic for 3 hours. Cells were stained with 50 nM TMRE for 20 min. 5 µM FCCP served as a positive control for destaining. TMRE fluorescence of viable cells was determined by flow cytometry.

*Ex vivo cytotoxicity assessment*

Primary ALL samples or ALL PDX cells were seeded at a density of 1x10^6^ cells/ml in SFEM II medium supplemented with 20% FCS, 1% Penicillin/Streptomycin and 20ng/ml human IL-7 and 10 ng/ml human IL-3 (PeproTech, Hamburg, Germany) on a feeder cell layer of human mesenchymal stem cells (MSC) as previously described (16). Cells were exposed to increasing concentrations of Dexamethasone, Dasatinib, Inotuzumab-ozogamicin, Venetoclax, or A1331852 alone or in combinations with fixed ratios (1:0.1:0.0067:1). After 48 hours, cells were stained with hCD19-APC (BioLegend, San Diego, US) and Calcein AM/propidium iodide (Invitrogen, Carlsbad, US/Serva, Heidelberg, Germany), and then assessed via flow cytometry.

*Animal experiments*

All animal studies were conducted in accordance with the German Animal Protection Law and the European Communities Council Directive 86/609/EEC and 2010/63/EU for the protection of animals used for experimental purposes. All experiments were approved by the Local Institutional Animal Care and Research Advisory Committee and permitted by the local authority, the Niedersächsische Landesamt für Verbraucherschutz und Lebensmittelsicherheit (No. 33.14-42502-04-19/3217, 33.12-42502-04-21/3711, and 33.19-42502-04-23-00289).

For treatment experiments 1x10^6^ ALL PDX cells were intravenously transplanted into the tail veins of female recipient NSG mice. Upon confirmed engraftment, as determined by in vivo bioluminescence imaging (BLI) IVIS or immunophenotyping of peripheral blood, mice were randomly allocated to each group and treated with the respective anti-neoplastic agents. Dexamethasone (1 mg/kg) and Dasatinib (10 mg/kg) were applied orally in 0.1 M sodium citrate by oral gavage 5 days per week in combination with either Venetoclax (20 mg/kg), A1331852 (20 mg/kg) or Navitoclax (20 mg/kg) in a vehicle consisting of 60% Phosal, 30% PEG 400 and 10% Ethanol with a treatment delay of minimum 2 hours 4 days per week. Tumor burden was assessed biweekly by peripheral blood monitoring or weekly by BLI using IVIS Lumina II after intraperitoneal application of XenoLight D-Luciferin (1mg/ mouse) (Perkin Elmer, Waltham, MA, US). To analyze the bioluminescence radiance, we used Living Image 4.7.4. After 4 weeks of oral gavage, mice were treated i.v. with Inotuzumab-ozogamicin (10 µg/kg) as consolidation therapy. Mice transplanted with the BCR::ABL1^+^ ALL PDX models received two applications of Inotuzumab over a period of one week. The lymphatic blast crisis PDX mice were treated 4 times over a period of 18 days.

*IC50 and Drug synergy*

Half-maximal inhibitory concentrations (IC50) and drug synergy were calculated using GraphPad Prism and CompuSyn software. The IC50 values were calculated and are stated for the Venetoclax or A1331852, respectively in the respective combination treatment. For drug synergy combination indices (CI) were calculated according to Chou-Talalay using CompySyn software. The CI values were calculated for the IC50 of the drug combination. CI values <1 were considered as synergistic interaction.

*Pretreatment short-term MOMP induction*

Ficoll-purified patient samples were cocultured in a density of 1x10^6^/ml on MSC feeder layers in SFEM II medium supplemented with 20% FCS 1% Penicillin/Streptomycin, 20 ng/ml IL-3 and 10 ng/ml IL-7. Samples were individually incubated with the respective personalized therapy: 200 nM Dexamethasone for 48 hours (#1), 50 ng/ml Inotuzumab for 24 hours (#2) or 200 nM Dexamethasone and 50 ng/ml Inotuzumab for 48 hours (#3). Drug concentrations were used that did not impair cell viability as monotherapy. After 24-48 hours coculture with or without pretreatment for mitochondrial priming, cells were incubated with 0.1 µM Venetoclax for 3 hours, followed by TMRE staining. Reduction of TMRE fluorescence by Venetoclax incubation without pretreatment was set as 1.

*Generation of ALL PDX models*

For the generation of ALL PDX models, isolated blast cells from patient samples were transplanted into the tail vein of 8- to 10-week-old NSG mice irradiated with 2.5 Gy. Leukemia engraftment was monitored by immunophenotyping of peripheral blood (huCD45, huCD19). Mice were sacrificed, and cells were isolated from the bone marrow and spleen and subsequently used for further experiments or transduced with the SLIEW lentiviral vector for stable expression of luciferase and GFP, as described earlier (17). Transduced cells were washed and further passaged in NSG mice. Isolated PDX cells were sorted for GFP expression (Sorter Facility MHH) and adoptively transplanted in NSG mice. L4967 and L707 cells (18) were initially provided by Olaf Heidenreich and expanded in our lab.

**Supplemental Tables:**

**Supplement Table 1: BCR::ABL1^-^ ALL patient characteristics.**

| **gender** | **age** | **immunophenotype** | **cytogenetics** |
| --- | --- | --- | --- |
| male | 20 | CD19+, CD10+, CD24+, CD15+ | t(12;21) |
| male | 19 | CD34+, CD20+, CD10+, cyTdT+, CD79a+, CD20+ | 46, XY |
| male |  | CD10+, CD20+, CD4+, CD24+, HLA-DR+, cyCD79a+, partial CD19+, CD38+ | n.a. |
| male | 85 | CD19+, CD10+, cyCD22+, CD24+, cyTdT+ | in 74% of interphase nuclei addition of Loci 3q26, 8q24, 22q11, 11q23 possibly due to hyperdiploidy |
| female | 17 | CD19+, CD10+, CD22+ | 48,XX,t(1;12)(q21;p13),del(6)(q14),?add(11)(q24),+12,+18,inc[4]/46,XX[11]. |
| female | 73 | CD19+, CD20+, cyCD79a+, CD24+, HLA-DR+, CD5+, CD10- | 42,XX,add(1)(p21),-8,add(9)(p21),-12,idic(13;14)(p11;p11),add(15)(p11),-17,add(18)(p11),inc[2]/46,XX,inc[3] |
| male | 39 | CD19+, CD10-, CD20-, CD22+ | 46,XY,t(4;11)(q21;q23) |
| male | 41 | CD19+, CD10-, CD20+, CD79a+, CD38+, CD34+ | 55,XY,+1,dup(4)(q22.1q22.1),+6,+8,inv(8)(q21.1q24),del(9)(p21.3),+10,+11q,+14,+18,+19,+21,+X |
| male | 37 | CD19+, CD10+, CD22+, CD24+, HLA-DR+ | 46,XY,der(1)t(1;19)(q23.3;p13.3),dup(1)(q23.3qter),der(6)t(6;7)(q12;q21.3)del(6)(q12qter),dup(7)(q21.3qter)del(9)(p13.2) |
| male | 37 | CD19+, CD10-, CD22+, HLA-DR+ | t(11;19)(q23;p13) |
| male | 77 | CD19+, no further information | n.a. |
| male | 67 | CD19+, CD10+, CD22+, CD24+, HLA-DR+, CD13+, cyTdT+, cyCD79a+ | 46, XY |
| female | 19 | CD10+, CD19+, CD20+, CD22+, HLA-DR+, CD13+, CD33+, cyTdT+ | no metaphases for analysis |
| female | 56 | CD34+, CD19+, CD10+, CD38+ | n.a. |
| male | 76 | CD19+, CD22+, HLA-DR+, cyTdT+, CD10- | 46,XY |
| male | 23 | CD34-, CD117-, CD19-, CD38+, CD79a+, CD22+ | n.a. |
| male | 63 | CD19+, CD10+ | n.a. |
| male | 40 | CD45+, CD10+, CD19+, CD22+, CD34-, CD38+, CD117-, HL-DR+, cyCD79a+, cyTdT- | n.a. |
| male | 73 | CD19+, CD22+, CD10+, HLA-DR+, CD24+, partial CD20+, CD15+, cyCD79a+ | 46,XY |
| female | 26 | CD19+, CD22+, CD10-, cyTdT+, CD22+, CD13+, CD123+, partial CD33+, CD38+, HLA-DR+ | 48,XX,t(7;12;9)(q35;p13;p12),t(9;15)(q33;q24),+12,+22[11]/46,XX[9] |
| male | 54 | CD34+, CD19+, CD33+ | n.a. |
| female | 33 | CD19+, CD22+, HLA-DR+, partial CD10+, CD13+, CD33+, CD123+, cyTdT+, cyCD79a+ | 46,XX,t(2;11)(p12;p13)[3]/46,XX |
| male | 55 | CD19+, CD15+, HLA-DR+ | complex aberrant karyotype |
| male | 20 | CD19+, CD10+, CD20-, CD22+, CD34+, CD24+, HLA-DR+, CD79a+, CD38+, CD123+, CD22+ | 53,XY,idem,+X,+Y,+der(1;5)t(1;5)(p11;q35),+6,-9,+10,der(19)t(19;20)(q13;q11),del(20)(q11),  +21,+21,+22[9]/46,XY,t(14;16)(q23;q24)[12] |
| male | 41 | CD19+, CD22+, CD34+, CD10+, cyTdT+, cyCD79a+ | 46,XY,del(9)(p21.3p21.3),del(9)(p21.3p21.3),del(13)(q14.2q14.2)/46,XY,idem,inv(X)(p11.3q23),inv(5)(q31.3q33.1),inv(7)(p12.2p12.1),inv(7)(q11.21q21.12),t(12;16;20)(p13.2;p13.3;q11.23) |
| male | 74 | CD19+, CD22+, no further information | n.a. |
| male | 49 | CD19+, partial CD20+, lowCD22, CD24+, CD38+, CD123+, lowCD13, HLA-DR+,cyTdT+, CD79a+ | 46,XY,del(2)(p16)x2,inv(9)(p12q13)c[2]/46,XY,inv(9)(p12q13)c[8] |
| male | 23 | CD34+, CD33+, CD19+, CD20+, CD10+, CD22+, cyTdT+ | n.a. |
| female | 54 | CD19+, CD20-, CD24+, CD38+, HLA-DR+, CD123low, CD22low | n.a. |
| male | 56 | CD34+, CD19+, cyTdT+, CD24+, CD38+, CD10-, CD20-, CD22- | n.a. |
| female | 39 | CD19+, CD34+ CD38+, CD24+, partial CD10+, CD22+ CD20- | 46,XX,del(9q),del(12q) |
| male | 35 | CD19+, cyCD79a+, CD10+, cyTdT+, HLA-DR+ | 64,X,-Y,+add(X)(q21),+del(X)(q21),+1,+5,  del(5)(q23q32),+6,+6,+8,+8,+10,+11,+12,+12,+14,+18,+18,+19,+21,+21,+22,+22[cp19]/46,XY[2] |
| male | 26 | CD19+, CD20+, CD10+ | n.a. |
| male | 51 | CD34+, CD19+, partial CD20+, CD10+, cyTdT+, CD22+, CD38+, HLA-DR+, TSLPR+ | n.a. |
| female | 80 | CD19+, CD22+, TSLPR+ | 46,XX,+20,+21,dic(20;21)(q11;p11)x2[15]/48,XX,+X,+21[6]/47,XX,del(8)(p21),t(9;22)(q34;q11),+21[2]/46,XX[2] |
| female | n.a. | CD34+, CD19+, CD10+ | n.a. |
| female | 66 | CD34-, CD19+, CD7-, cyCD79a+, CD10-, CD20-, CD38+, cyTdT+, CD33+ | n.a. |
| female | 59 | CD19+, CD10+, CD24+, CD13+, cyTdT+, MPO-, CD117- | 45, XX, t(1;16)(q21;22),-7,t(9;22)(q34;q11) |

**Supplement Table 2: BCR::ABL1^+^ ALL patient characteristics.**

| **gender** | **age** | **immunophenotype** | **cytogenetics** | **molecular genetics** |
| --- | --- | --- | --- | --- |
| female | 59 | CD19+, CD10+, CD24+, CD13+, cyTdT+, MPO-, CD117- | 45, XX, t(1;16)(q21;22),-7, t(9;22)(q34;q111) | BCR::ABL1+, p190 |
| female | 19 | CD34+, CD19+, CD33+ | t(9;22)(q34;q11) | BCR::ABL1+, p190 |
| male | 49 | CD19+, CD10+, HLA-DR+, cyTdT+, CD24+, partial CD20+ | t(9;22)(q34;q11) | BCR::ABL1+, p210 |
| female | 47 | CD19+, CD10+, CD20+, CD22+, CD24+, HLA-DR+, cyTdT+, cyCD79a+ | 46,XX, t(9;22)(q34;q11) | BCR::ABL1+, p190 |
| female | 73 | CD19+, CD10+, CD24+, HLA-DR+, CD13+, CD33+, CD20- | t(9;22)(q34;q11) | BCR::ABL1+, p210 |
| male | 73 | CD19+, CD24+, CD20+, CD10+, cyTdT+, HLA-DR+ | 46,XY,t(9;22)(q34;q11),i(9)(q10) | BCR::ABL1+, p210 |
| female | 59 | CD19+, CD10+, CD24+, cyTdT+, HLA-DR+, lowCD20 | t(9;22)(q34;q11) | BCR::ABL1+, p210 |
| female | 38 | CD19+, CD10+, CD20+, CD22+, cyTdT+, CD13+, CD15+ | t(9;22)(q34;q11) | BCR::ABL1+, p190 |
| male | 70 | CD19+, CD10+, CD24+, HLA-DR+, CD34+, CD20+, CD22+, CD13+, CD15+ | 45,XY,-9,t(9;22)(q34;q11) | BCR::ABL1+, p190 |
| male | 35 | CD34+, CD19+, CD22-, CD10+, CD20-, | t(9;22)(q34;q11) | BCR::ABL1+ |
| female | 68 | CD19+, CD10+, CD22+, CD24+, HLA-DR+, cyTdT+, cyCD79a+, CD20+ | 51,XX,+X,+X,+4,del(7)(p12p15),add(9)(p12),t(9;22)(q34;q11),+14,+der(22)t(9;22) | BCR::ABL1+ |
| male | 40 | CD34+, CD19+, CD10+, CD24+, CD123+, HLA-DR+, cyTdT+, cyCD79a+ | 46-48 XY, t(9;22), +8, del(8)(p11), der(8;12)x1-2,i(8)(q10), +12, +17 | BCR::ABL1+, p210, ABL-kinase mutation c.703A>G; p.Met244Val |
| female | 59 | CD19+, CD10+, CD24+, cyTdT+, cyCD79a+, CD20+, CD13+, CD33+, CD14+, HLA-DR+, CD123+ | 46,XX,t(9;22)(q34;q11)[3]/50,idem,+X,+X,+5,der(22)t(9;22)[6]/46,XX[6] | BCR::ABL1+, p210 b2a2 |
| female | 58 | CD19+, CD10+, CD22+ | t(9;22)(q34;q11) | BCR::ABL1+ |
| male | 69 | CD19+, CD10+, CD20+, CD22+ | t(9;22)(q34;q11) | BCR::ABL1+ |
| female | 80 | CD19+, CD10+, CD22+, CD24+, CD123+, lowCD13, CD38+, CD20-, HLA-DR+ | t(9;22)(q34;q11) | BCR::ABL1+ |

**Supplemental Figures:**

**
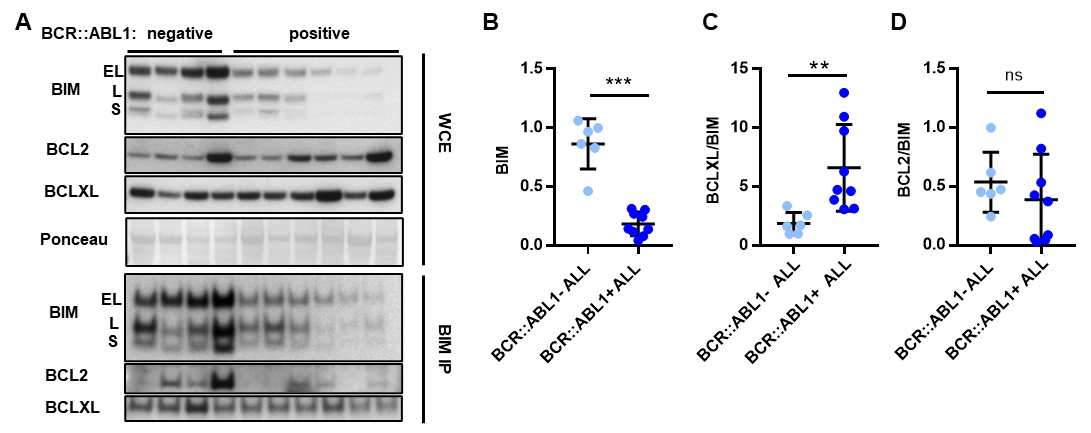
**

**Supplemental Figure 1:** **A)** Representative BIM-immunoprecipitation and whole cell extracts (WCE) from primary PDX BCR::ABL1^-^ or BCR::ABL1^+^ ALL samples. **B-D)** Densitometric analysis of BIM-immunoprecipitations of n=6 primary PDX BCR::ABL1^-^ and n=9 BCR::ABL1^+^ ALL samples. **p<0.01, ***p<0.001 indicate statistical significance assessed by Student´s t-test.


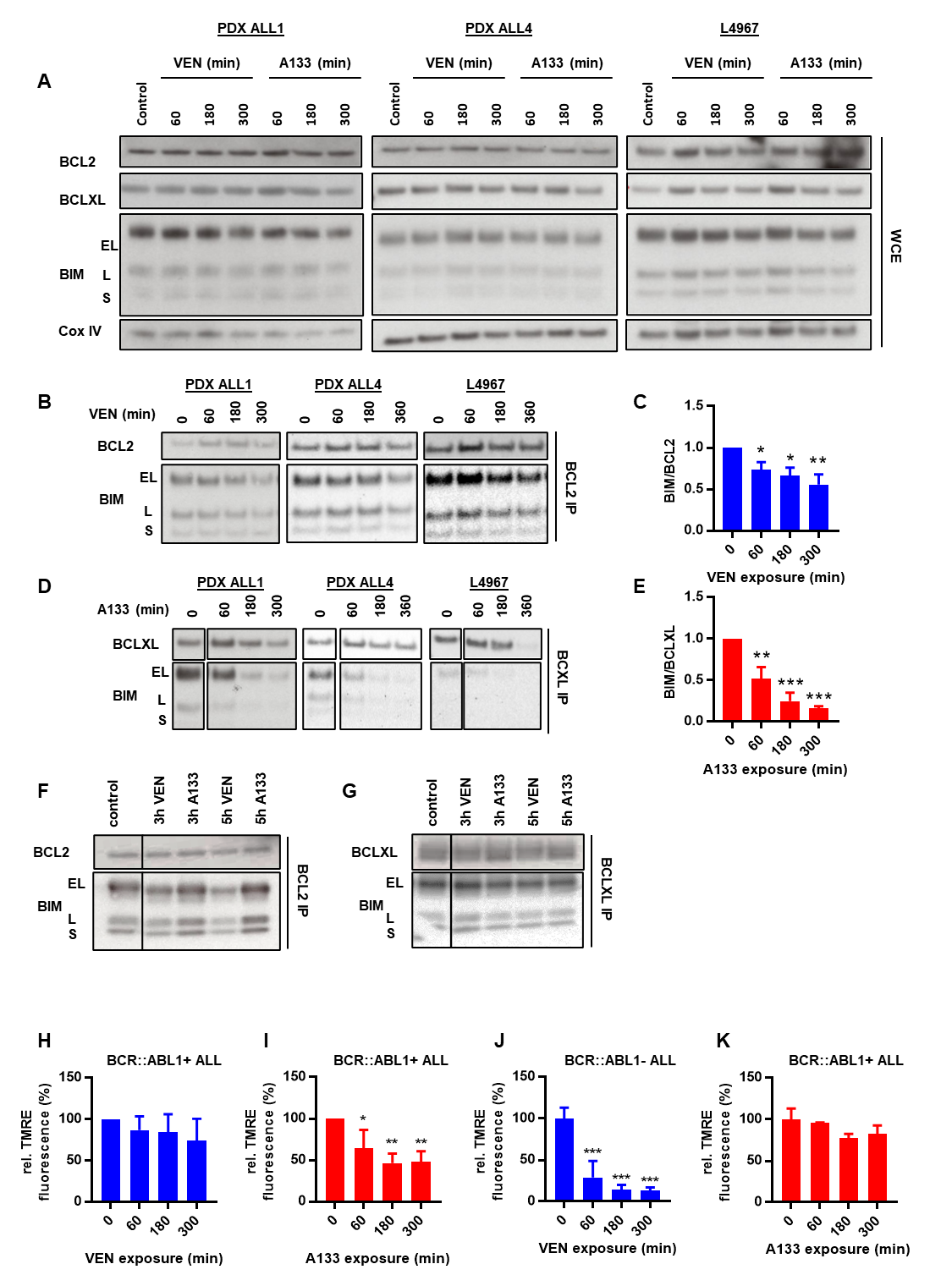


**Supplemental Figure 2:** **A)** Immunoblots (whole cell extracts) of PDX ALL1(left), PDX ALL4 (middle) and L4967 cells (right) treated for 1, 3 and 5 hours with 1µM Venetoclax and 1µM A1331852. Cox IV served as loading control. **B)** BCL2-immunoprecipitations and **C)** densitometric analysis of BIM/BCL2 of three BCR::ABL1^+^ ALL PDX models (PDX ALL1, ALL4, L4967) after 1, 3 and 5 hours Venetoclax (1µM). Samples were immunoblotted and incubated with detection antibodies for BCL2, BCLXL and BIM, respectively. **D)** BCLXL-immunoprecipitations and **E)** densitometric analysis of BIM/BCLXL of three BCR::ABL1^+^ ALL PDX models (PDX ALL1, ALL4, L4967) after 1, 3 and 5 hours A1331852 (1µM). **F)** BCL2- and **G)** BCLXL-immunoprecipitation of RS4;11 cells treated for 3 and 5 hours with 1 µM Venetoclax and 1µM A1331852, respectively. **H-I)** TMRE staining of PDX ALL1, PDX ALL4 and L4967 cells treated for 1, 3 and 5 hours with 1µM Venetoclax and 1µM A1331852, respectively. **J, K)** TMRE staining of RS4;11 cells treated for 3 and 5 hours with 1µM Venetoclax and 1µM A1331852, respectively. *p<0.05, **p<0.01, ***p<0.001 indicate statistical significance assessed by one-way ANOVA with Bonferroni post-hoc test.


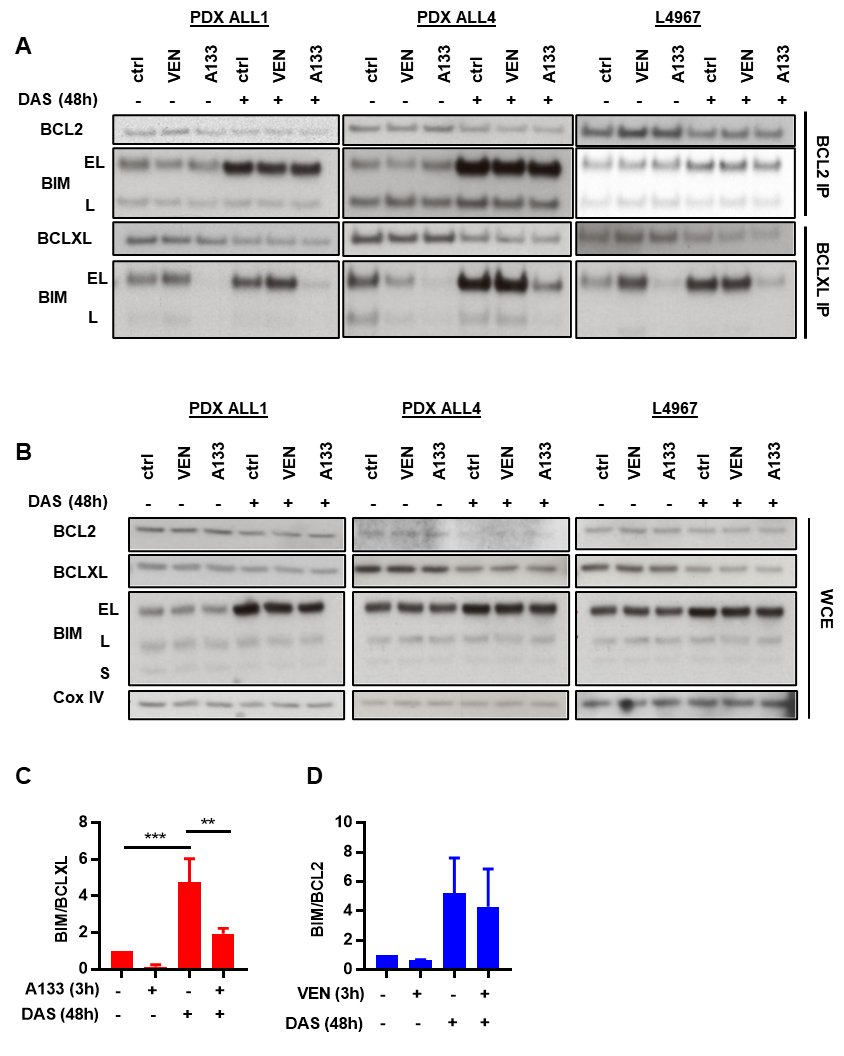


**Supplemental Figure 3: A)** BCL2- and BCLXL-immunoprecipitations and **B)** whole cell extracts of PDX ALL1, PDX ALL4 and L4967 cells treated for 48 hours with 50nM Dasatinib, followed by short-term treatment with Venetoclax (1µM) or A1331852 (100nM) for 3 hours. Cox IV served as loading control. **C-D**) Respective densitometric analysis of BIM/BCLXL and BIM/BCL2 in immunoprecipitations of PDX ALL1, PDX ALL4 and L4967 cells (mean). **p<0.01, ***p<0.001 indicate statistical significance assessed by one-way ANOVA with Bonferroni post-hoc test.


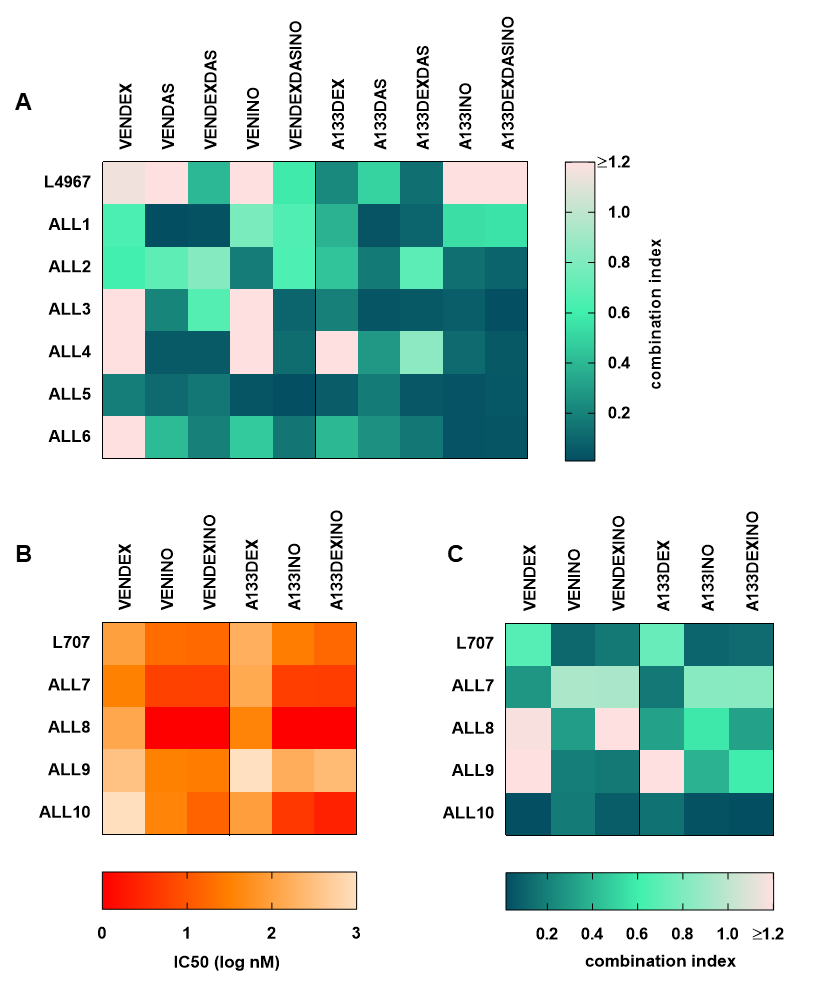


**Supplemental Figure 4: A)** Combination indices (CI) at the respective IC50 of drug combination therapies in BCR::ABL1^+^ ALL primary or PDX samples. **B)** Cytotoxicity assessment (IC50) and **C)** CI of combination therapies in BCR::ABL1^-^ primary or PDX samples ex vivo on MSC feeder layers after 48 hours. B) Calculated IC50 (log nM) of the BH3 mimetic concentration in the respective combinations and C) CI values at the respective IC50 are plotted. CI<1 indicates drug synergy. Drugs were used in fixed ratios of Venetoclax/A1331852:Dexamethasone:Inotuzumab 1:1:0.0067.

**
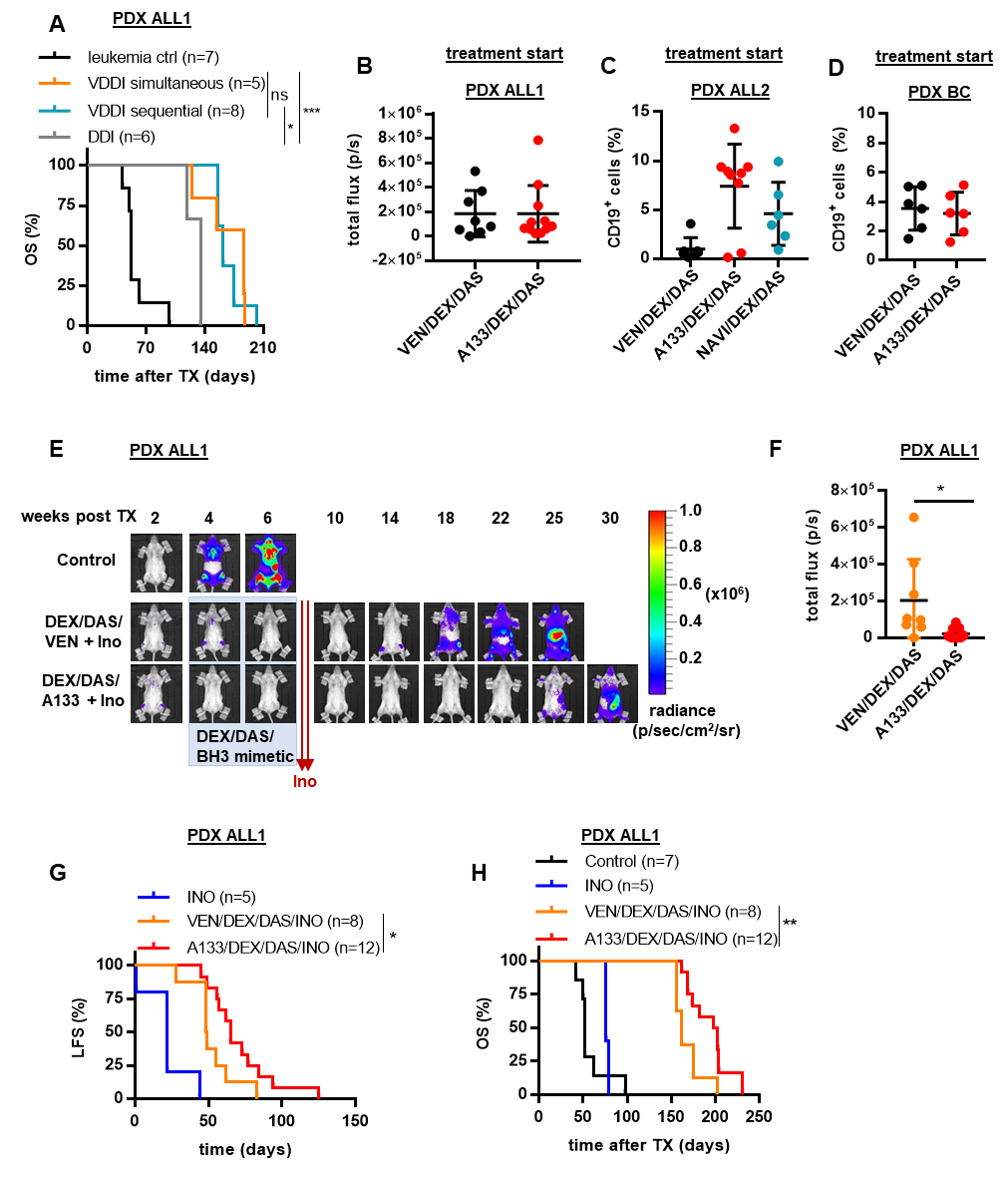
**

**Supplemental Figure 5: A)** Survival of NSG mice engrafted with luciferase-transduced PDX ALL1 cells. Mice were treated either with the combination DEX/DAS/INO (DDI) or with VEN/DEX/DAS/INO (VDDI). In the VEN/DEX/DAS/INO group, INO was administered either during the combination treatment period (simultaneous) or afterward (sequential). **B)** Total flux of PDX ALL1 mice at the start of treatment. **C, D)** Proportion of human CD19^+^ cells in peripheral blood at start of treatment for C) PDX ALL2 and D) PDX BC mice. **E)** Representative in vivo bioluminescence imaging (BLI) of PDX ALL1 mice and **F)** quantification of total flux of BLI from PDX ALL1 mice 2 weeks after start of oral treatment with either VEN/DEX/DAS or A133/DEX/DAS. **G)** Leukemia-free survival (LFS) and **H)** overall survival (OS) of PDX ALL1 mice treated with INO alone, or with the combinations VEN/DEX/DAS/INO or A133/DEX/DAS/INO, respectively. *p<0.05, **p<0.01, ***p<0.001 indicate statistical significance assessed by Student´s t-test (F) or log-rank test (A, G, and H).


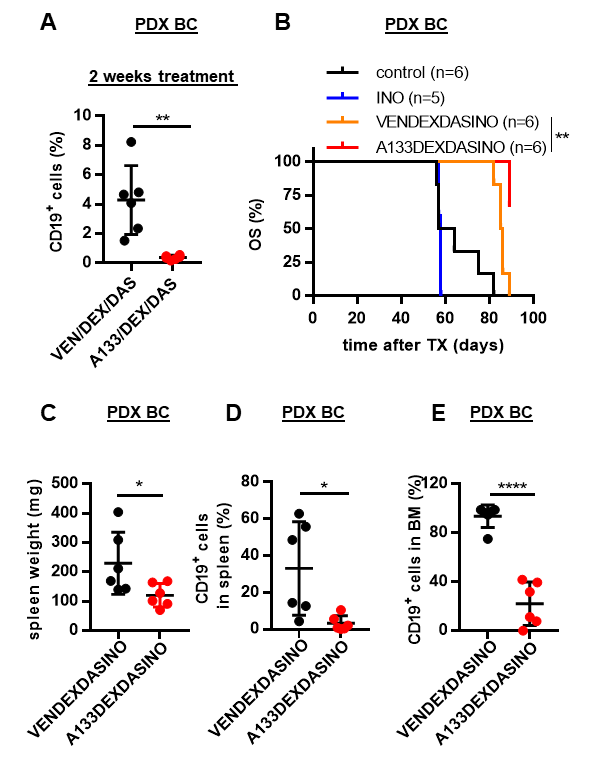


**Supplemental Figure 6: A)** Proportion of human CD19^+^ cells in PB of PDX BC mice 2 weeks after treatment start with VEN/DEX/DAS and A133/DEX/DAS, respectively. **B)** Survival of PDX BC mice treated with INO, VENDEXDASINO, or A133DEXDASINO. **C-E)** Post-mortem analysis of PDX BC mice for C) spleen weight, D) CD19^+^ cells in spleen, and E) CD19^+^ cells in bone marrow (BM). *p<0.05, **p<0.01, ****p<0.0001 indicate statistical significance assessed by Student´s t-test (A, C-E) or log-rank test (B).

**
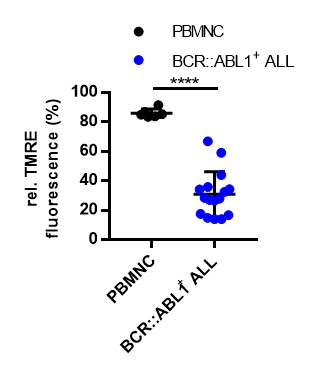
**

**Supplemental Figure 7:** Flow cytometric analysis of TMRE fluorescence of PBMNC from healthy donors (n=6) and primary BCR::ABL1^+^ ALL blasts (n=16) treated with 1µM Navitoclax for 3 hours and stained with TMRE. ****p<0.0001 indicates statistical significance assessed by Student´s t-test.


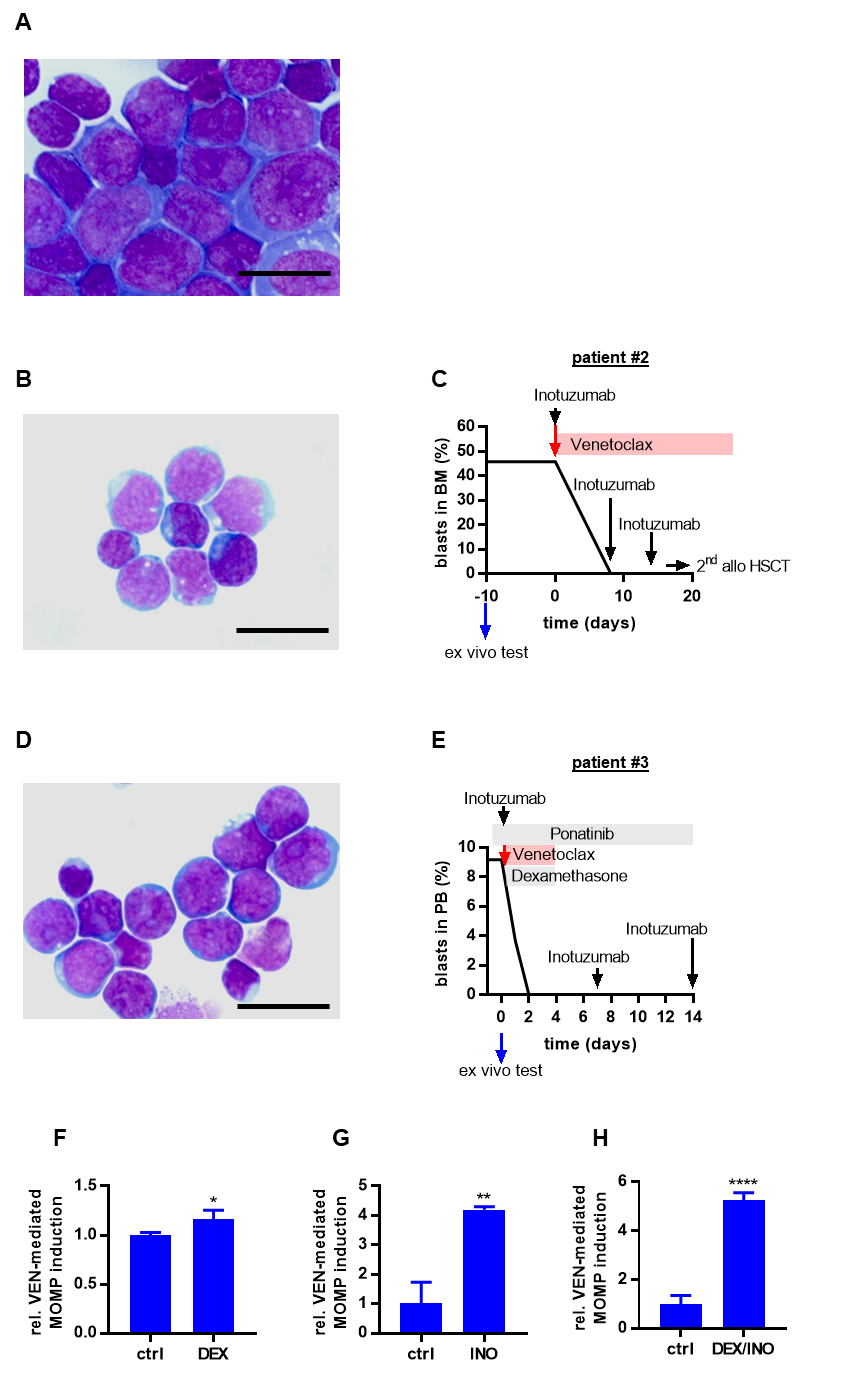


**Supplemental Figure 8:** **A)** Cytospin with May-Grünwald-Giemsa staining of Ascites (1:300 diluted) from patient #1 with r/r BCR::ABL1^-^ ALL. Scale bar: 20µM, **B)** Cytospin with May-Grünwald-Giemsa staining of ficoll-purified cells from bone marrow of patient #2 with r/r BCR::ABL1^-^ ALL. Scale bar: 20µM. **C)** Therapy schedule and clinical response of patient #2. **D)** Cytospin with May-Grünwald-Giemsa staining of ficoll-purified cells from PB of patient #3 with CML lymphoid blast crisis. Scale bar: 20µM. **E)** Therapy schedule and clinical response of patient #3. **F-H)** TMRE staining of patient samples cocultured on MSC feeder layers and treated with F) 200nM Dexamethasone for 48 hours, G) 50ng/ml Inotuzumab for 24 hours or H) 200nM Dexamethasone and 50ng/ml Inotuzumab for 48 hours. Subsequently cells were treated with or without 0.1µM Venetoclax for 3 hours and TMRE staining was performed. Reduction of TMRE fluorescence by Venetoclax incubation in cells without pretreatment was set as 1. *p<0.05, **p<0.01, ****p<0.0001 indicate statistical significance assessed by Student´s t-test.

**Supplemental References**

16. Kirchhoff H, Karsli U, Schoenherr C, Battmer K, Erschow S, Talbot SR, et al. Venetoclax and dexamethasone synergize with inotuzumab-ozogamicin induced DNA damage signaling in B-lineage ALL. Blood. 2021;137(19):2657–61.

17. Bomken S, Buechler L, Rehe K, Ponthan F, Elder A, Blair H, et al. Lentiviral marking of patient-derived acute lymphoblastic leukaemic cells allows in vivo tracking of disease progression. Leukemia. 2013;27(3):718-21.

18. Rehe K, Wilson K, Bomken S, Williamson D, Irving J, den Boer ML, et al. Acute B lymphoblastic leukaemia-propagating cells are present at high frequency in diverse lymphoblast populations. EMBO Mol Med. 2013;5(1):38-51.
